# Mitochondrial ATP Depletion Disrupts Caco-2 Monolayer Integrity and Internalizes Claudin 7

**DOI:** 10.3389/fphys.2017.00794

**Published:** 2017-10-11

**Authors:** Lonneke M. JanssenDuijghuijsen, Sander Grefte, Vincent C. J. de Boer, Lara Zeper, Dorien A. M. van Dartel, Inge van der Stelt, Melissa Bekkenkamp-Grovenstein, Klaske van Norren, Harry J. Wichers, Jaap Keijer

**Affiliations:** ^1^Wageningen Food and Biobased Research, Wageningen University and Research, Wageningen, Netherlands; ^2^Human and Animal Physiology, Wageningen University and Research, Wageningen, Netherlands; ^3^Division of Human Nutrition, Wageningen University and Research, Wageningen, Netherlands; ^4^Nutricia Research, Utrecht, Netherlands

**Keywords:** Caco-2, permeability, mitochondria, intestinal epithelium, galactose, tight junctions

## Abstract

**Objective:** *In vivo* studies suggest that intestinal barrier integrity is dependent on mitochondrial ATP production. Here, we aim to provide mechanistic support, using an *in vitro* model mimicking the oxidative *in vivo* situation.

**Methods:** Human Caco-2 cells were cultured for 10 days in culture flasks or for 14 days on transwell inserts in either glucose-containing or galactose-containing medium. Mitochondria were visualized and cellular respiration and levels of oxidative phosphorylation (OXPHOS) proteins were determined. Mitochondrial ATP depletion was induced using CCCP, rotenone, or piericidin A (PA). Monolayer permeability was assessed using transepithelial electrical resistance (TEER) and fluorescein flux. Gene expression and cellular distribution of tight junction proteins were analyzed.

**Results:** Caco-2 cells cultured in galactose-containing, but not in glucose-containing, medium showed increased mitochondrial connectivity, oxygen consumption rates and levels of OXPHOS proteins. Inhibition of mitochondrial ATP production using CCCP, rotenone or PA resulted in a dose-dependent increase in Caco-2 monolayer permeability. In-depth studies with PA showed a six fold decrease in cellular ATP and revealed increased gene expression of tight junction proteins (*TJP*) 1 and 2, occludin, and claudin 1, but decreased gene expression of claudin 2 and 7. Of these, claudin 7 was clearly redistributed from the cellular membrane into the cytoplasm, while the others were not (TJP1, occludin) or slightly (claudin 2, actin) affected. *In vivo* studies suggest that intestinal barrier integrity is dependent on mitochondrial ATP production. Here, we aim to provide mechanistic support, using an *in vitro* model mimicking the oxidative *in vivo* situation.

**Conclusions:** Well-functioning mitochondria are essential for maintaining cellular energy status and monolayer integrity of galactose grown Caco-2 cells. Energy depletion-induced Caco-2 monolayer permeability may be facilitated by changes in the distribution of claudin 7.

## Introduction

The gut is the major organ at the interface of the internal and external environment and plays an important role in nutrient uptake and metabolism, metabolic homeostasis and health. The splanchnic region is highly vascularized, which is pivotal to support the high metabolic demands of proper gut functioning (Rolfe and Brown, 1997). The enterocytes lining the gut barrier are very sensitive to ischemic conditions (Hinnebusch et al., 2002). Intestinal ischemia results in a state of inflammation and increased intestinal permeability (Grotz et al., 1999; Grootjans et al., 2010). This suggests that oxidative energy metabolism plays an essential role in maintaining a gut barrier with high integrity. Several *in vivo* studies indirectly support this hypothesis, such as exercise studies that show an exercise-induced splanchnic hypoperfusion (Van Wijck et al., 2011) and exercise-induced intestinal permeability (Van Wijck et al., 2011; JanssenDuijghuijsen et al., 2016, 2017). Fructooligosaccharide supplementation in rats furthermore resulted in an increase in intestinal permeability (Rodenburg et al., 2008). Although intestinal cellular energy status was not assessed in this study, mitochondrial genes were highly regulated in a way similar to that induced by mitochondrial uncouplers. Finally, non-steroid anti-inflammatory drugs are thought to induce intestinal permeability at least in part by mitochondrial uncoupling (Mahmud et al., 1996; Somasundaram et al., 2000; Bjarnason and Takeuchi, 2009). These findings together suggest an essential role for mitochondrial ATP production in maintaining a gut barrier with high integrity.

The Caco-2 cell line can be used to study the direct relation between mitochondrial ATP production and intestinal permeability. This human intestinal epithelial cell model, derived from a colon adenocarcinoma, demonstrates many *in vivo* intestinal functions and is the most widely used model to study the epithelial barrier function (Sambuy et al., 2005). Good correlations have been reported between *in vitro* Caco-2 cell permeability and *in vivo* absorption in humans (Yee, 1997). Caco-2 cells, however, are routinely cultured in high glucose conditions and thereby rely mainly on glycolysis for their ATP production. For several cell types it has been shown that an *in vitro* energy substrate switch from glucose to galactose results in a cellular phenotype relying more on mitochondrial ATP production (Rossignol et al., 2004; Marroquin et al., 2007; Aguer et al., 2011; Kase et al., 2013). Such an *in vitro* substrate switch induces an oxidative phenotype and provides an improved representation of the *in vivo* situation which enables to study the role of mitochondrial ATP production in maintaining a healthy gut barrier. Therefore, our aim was to develop an *in vitro* intestinal permeability model mimicking the *in vivo* oxidative intestinal phenotype and, more crucially, to determine the dependency on mitochondrial ATP production of intestinal barrier integrity.

## Materials and methods

### Caco-2 cell culture

The Caco-2 human colon cancer cell line was obtained from the American Type Culture Collection (ATCC HTB-37TM, Manassas, VA, USA). These cells were routinely grown in 75 cm^2^ culture flasks (Corning, Amsterdam, NL) in high-glucose (25 mM) Dulbecco's Modified Eagle's medium (DMEM-glucose, Gibco, Breda, NL) supplemented with 100 U/mL penicillin and 100 μg/mL streptomycin (Gibco), 1 mM Na-pyruvate (Gibco), 2 mM glutamax (Gibco), 25 mM HEPES buffer (Gibco) and 10% heat-inactivated (56°C, 45 min) fetal bovine serum (FBS, HyClone, Thermo Scientific, Breda, NL). Caco-2 cells were cultured at 37°C in a humidified atmosphere with 5% CO_2_ up to 80% confluency and then either subcultured or used for the experiments. For all experiments, Caco-2 cells with a passage number between 30 and 36 were proliferated or differentiated using either complete supplemented DMEM-glucose or complete supplemented DMEM-galactose [(= glucose-free DMEM (GIBCO) supplemented with 25 mM galactose as well as the other supplements as in DMEM-glucose)].

### Mitochondrial respiration of Caco-2 cells

Proliferating Caco-2 cells were cultured in DMEM-glucose or DMEM-galactose for 10 days after which oxygen consumption rate (OCR) was measured using 1.0·10^6^ cells. OCR was measured at 37°C using polygraphic oxygen sensors in a two-chamber Oxygraph (Oroboros Instruments, Innsbruck, AT). Basal OCR was determined first followed by (1) leak OCR: 1 μM oligomycin (Sigma Aldrich), blocking complex V of the OXPHOS; (2) maximal OCR: titration with 1 μM carbonyl cyanide m-chlorophenylhydrazone (CCCP, Abcam Cambridge, UK), uncoupling OXPHOS; (3) non-mitochondrial OCR: 1 μM antimycin A (Sigma Aldrich) and 1 μM rotenone (ROT, Sigma Aldrich), blocking complex III and I, respectively. This experiment was performed six times.

Caco-2 cells (1.0·10^5^ cells/cm^2^) were also cultured in the XF96 cell culture microplate using DMEM-glucose or DMEM-galactose for 14 days to induce (semi-)differentiation. OCR and ECAR were measured with the XFe96 Extracellular Flux Analyser (Seahorse Bioscience, Billerica, MA, USA). Basal OCR and ECAR were determined first, followed by leak OCR and maximal ECAR: 15 μM oligomycin; maximal OCR: 1 μM carbonyl cyanide-p-trifluoromethoxyphenylhydrazone (FCCP, Sigma Aldrich); non-mitochondrial OCR: 2.5 μM antimycin A and 1.25 μM ROT. The FCCP concentration used was obtained from a titration experiment. The basal, leak, and maximal OCR values were corrected for non-mitochondrial OCR. OCR and ECAR values were normalized by cellular protein content, as analyzed with the Bio-Rad DC colorimetric protein assay (Bio-Rad laboratories, Veenendaal, NL).

### Mitochondrial visualization in proliferating Caco-2 cells

To visualize the mitochondria, Caco-2 cells were seeded in 35 mm plastic dishes (Eppendorf, Nijmegen, NL) at 0.3–1.0 × 10^4^ cells per cm^2^ in DMEM-glucose or DMEM-galactose and cultured for 10 days when 70% confluency was reached. Then, culture medium was replaced by phenol red-free DMEM (Similarly supplemented as DMEM-glucose or DMEM-galactose) with 200 nM MitoTracker Green FM (Life Technologies, Breda, NL). After 30 min of incubation, the MitoTracker-containing medium was replaced by fresh DMEM-glucose or DMEM-galactose and mitochondrial morphology was visualized with the EVOS FL Color Imaging System (Life Technologies).

### Caco-2 cell differentiation and polarization

To induce differentiation and polarization, Caco-2 cells were seeded at a density of 1.0·10^5^ cells/cm^2^ in 24-well transwell inserts (0.4 μM diameter ThinCerts, Greiner Bio-one, Alphen a/d Rijn, NL) and differentiated for 14 days in DMEM-glucose or DMEM-galactose. Culture medium was changed three times a week and transepithelial electrical resistance (TEER) was monitored using a MilliCell-ERS voltohmmeter (Millipore, Etten-Leur, NL). Only the monolayers with TEER values exceeding 200 Ω·cm^−2^ were used for Caco-2 monolayer permeability experiments and for determination of OXPHOS protein levels and qPCR gene expression analysis.

### Western blotting-OXPHOS protein levels

Fourteen-days differentiated Caco-2 cells were washed with ice-cold HBSS, lysed, and sonicated in 50 mM TRIS-HCl (pH 7.4) supplemented with 1% Triton (Sigma Aldrich) and 10% protease inhibitor (P8340, Sigma Aldrich). Experiments were performed in triplicate. Protein concentrations were determined with the Bio-Rad DC colorimetric protein assay (Bio-Rad laboratories) according to manufacturer's protocol. Protein samples were heated at 50°C for 5 min, separated (30 μg protein per sample per lane) by SDS-PAGE using 12% polyacrylamide gels and finally transferred to an Immobilon membrane (Millipore). Five individual proteins representing the five different OXPHOS complexes (NDUFB8 for Complex I; SDHB for Complex II; UQCRC2 for Complex III; COX II for Complex IV; ATP5A for Complex V) were detected (incubation overnight at 4°C) with total anti-OXPHOS human antibody cocktail (1:1,000, Ab110411, Abcam). Anti-β-actin antibody (1:1,000, Ab8227) was used as a loading control. Bound primary antibodies were visualized using IRDye 800CW goat anti-mouse IgG secondary antibody (1:10,000; LI-COR Biosciences Inc., Lincoln, NE, USA). Blots were scanned with LI-COR's Odyssey Infrared Image System.

### Caco-2 monolayer permeability experiments

Fourteen-days differentiated Caco-2 cells were used for monolayer permeability experiments, which were all performed in triplicate. Caco-2 cells were incubated with varying concentrations of either vehicle (96% EtOH), CCCP (mitochondrial uncoupler, 0-1-2-3-4-5 μM), rotenone (ROT, Complex I inhibitor, 0-50-100-200 nM), and Piericidin A (PA, Complex I inhibitor, 0-50-100-200 nM; Cayman Chemicals, Ann Arbor, MI, USA) for 27, 30, and 42 h, respectively. Changes in TEER were followed over time to determine permeability of the Caco-2 monolayer. Apical medium was harvested to determine cytotoxicity with the Pierce LDH Cytotoxicity assay kit (Thermo Scientific), according to the manufacturer's instructions.

One hundred μg/mL fluorescein (Sigma Aldrich) was added to the apical medium of Caco-2 cells incubated with vehicle (96% EtOH) or 200 nM PA for 24 h prior to harvesting the basolateral medium at different time points. Caco-2 cells incubated with vehicle (96% EtOH) or 200 nM PA were also harvested at different time points to determine cellular energy status and to isolate RNA for gene expression analysis.

### HPLC-cellular energy status of Caco-2 cells

Fourteen-days differentiated Caco-2 cells were incubated with vehicle (96% EtOH) or 200 nM PA and then harvested at several time points to measure cellular ATP levels with high pressure liquid chromatography analysis, adapted from Di Pierro et al. (1995). All samples were deproteinized by mixing with 100 μL 7% perchloric acid, followed by desalting with 125 μL 1 M KOH, neutralizing with 75 μL 0.1 M phosphate buffer pH 6.5, and centrifugating for 10 min at 14,000 g at 4°C. Supernatant was snap-frozen in liquid nitrogen and stored at −80°C until further analysis. Parallel cultured samples were harvested to determine protein content to correct ATP measurements. Protein content was determined with the Bio-Rad DC colorimetric protein assay (Bio-Rad laboratories) according to manufacturer's protocol. Twenty-five microliter of the supernatant was analyzed by HPLC (LaChrom Elite, Merck-Hitachi) with UV detection at 254 nm (Kratos). The chromatography was performed on an HPLC C18 column (Aqua; 150 × 4.6 mm, 5 μm, Phenomenex) using a gradient elution with two mobile phases. Mobile phase A consisted of 0.1 M phosphate buffer pH 6.0 and mobile phase B consisted of 0.1 M phosphate buffer pH 6.0/methanol (60/40). The gradient, at a flow rate of 1 ml/min, was as follows: 0–5 min isocratic at 0% mobile phase B; 5–16 min linear gradient from 0 to 22% B; 16–25 min linear gradient from 22 to 60% B; 25–27 min linear gradient from 60 to 100% B; 27–28 min isocratic at 100% B; 28–29 min linear return from 100 to 0% B; 29–35 min isocratic at 0% B, making a total run time of 35 min. Peaks were integrated with Clarity Lite software (DataApex, Prague, Czech Republic) and cellular ATP levels were calculated using calibration curves made with a commercial standard (Sigma Aldrich), and corrected for protein content.

### qPCR- gene expression analysis

Total cellular RNA was isolated from 14-days differentiated Caco-2 cells incubated with vehicle (96% EtOH) or 200 nM PA when TEER was high or low using the Qiagen RNeasy Kit (Qiagen, Westburg, Leusden, NL) in combination with the on-column DNase I (Bio-Rad) treatment, according to the manufacturer's instructions. RNA quantity was checked with the Nanodrop spectrophotometer (IsoGen Life Science, Maarssen, NL) and RNA integrity was checked by capillary zone electrophoresis (Experion, Bio-Rad). RNA of all individual samples (*n* = 6 per condition) was reverse transcribed using the iScript cDNA synthesis kit (Bio-Rad). Gene expression measurements were performed in a 25 μL reaction mix containing iQ SYBR Green Supermix (Bio-Rad) using the CFX96 Real-Time System (Bio-Rad). Target and reference genes, gene symbols and primer sequences are shown in Table 1. Reference genes were selected based on stability. The expression of the gene of interest was normalized against the geometrical mean of the reference genes with the CFX software (Bio-Rad).

**Table 1:** Primer sequences of reference genes and genes of interest.

	**Abbreviations**	**Forward primer 5′−3′**	**Reverse primer 5′−3′**
**REFERENCE GENES**			
β-actin	*ACTB*	CTGGAACGGTGAAGGTGACA	AAGGGACTTCCTGTAACAATGCA
β-2 microglobulin	*B2M*	TGCCGTGTGAACCATGTG	GCGGCATCTTCAAACCTC
Transmembrane protein 14C	*TMEM14C*	CCGCTTGTTTTCTGCAGGTG	CACGCTGCCTGCTTTTACATAG
Glutaminyl-tRNA synthetase	*QARS*	TAAGTGACCTGAACCTGGCATC	GACGCTCAAACTGGAACTTGTC
**GENES OF INTEREST**			
Sucrase-isomaltase	*SIM*	GAGGACACTGGCTTGGAGAC	ATCCAGCGGGTACAGAGATG
Tight junction protein 1	*TJP1*	GGGAACAACATACAGTGACGC	CCCCACTCTGAAAATGAGGA
Tight junction protein 2	*TJP2*	GGAGGATGTGCTTCATTCG	GGCCTCTTGACCACAATAG
Occludin	*OCLN*	CCCATCTGACTATGTGGAAAGA	AAAACCGCTTGTCATTCACTTTG
Claudin 1	*CLDN1*	TTTCCTGCTACAACAATCCTCTCC	GTTGTTTTTCGGGGACAGGAAC
Claudin 2	*CLDN2*	CTCCCTGGCCTGCATTATCTC	ACCTGCTACCGCCACTCTGT
Claudin 7	*CLDN7*	CTGCAAAATGTACGACTCGGTG	GCAAGACCTGCCACGATGAAAA

### Immunofluorescent staining of tight junction proteins

Fourteen days-differentiated Caco-2 cells incubated with vehicle (96% EtOH) or 200 nM PA were fixed in 4% formaldehyde at time points when TEER was high or low. To visualize tight junction protein 1 (TJP1; also known as ZO-1), occludin (OCLN) and claudin 7 (CLDN7), Caco-2 cells were first permeabilized with 0,5% (v/v) Tween-20 in PBS for 20 min and then blocked in blocking buffer containing 2% (w/v) bovine serum albumin (BSA), 0.2% (v/v) horse serum, 0.5% (v/v) Tween-20, and 100 mM glycine in PBS for 30 min. This was followed by a 90-min incubation with the primary rabbit antibodies against TJP1 (40–2,200, 1:100, Thermo Fischer Scientific, Rockford, MA, USA), OCLN (ab31721, 1:1,000, Abcam, Cambridge, UK), and CLDN7 (ab203700, 1:250, Abcam, Cambridge, UK). Bound primary antibodies were visualized with Alexa Fluor-488-labeled goat-anti-rabbit IgG (H+L) (1:200, Molecular probes). CytoPainter Phalloidin-iFluor 594 (1:1,000, Abcam) was included to visualize actin filaments. Caco-2 cells were finally incubated with 4′,6-diamidino-2-phenylindole (DAPI; 1 μg/ml) to stain all nuclei and sealed using Fluoromount G (Southern Biotech, Birmingham, USA). To visualize claudin 2 (CLDN2), Caco-2 cells were permeabilized with 0,5% (v/v) Triton X-100 in PBS for 30 min and then blocked in blocking buffer containing 2% (w/v) BSA, 0.2% (v/v) goat serum, 0.1% (v/v) Triton X-100 and 0.05% (v/v) Tween-20 in PBS for 20 min. This was followed by 90-min incubation with the rabbit primary antibody against CLDN2 (ab53032, 1:100, Abcam, Cambridge, UK). Further steps were similar to TJP1, OCLN and CLDN7.

### Localization and distribution analysis by leica microscope

**Figure 1:**
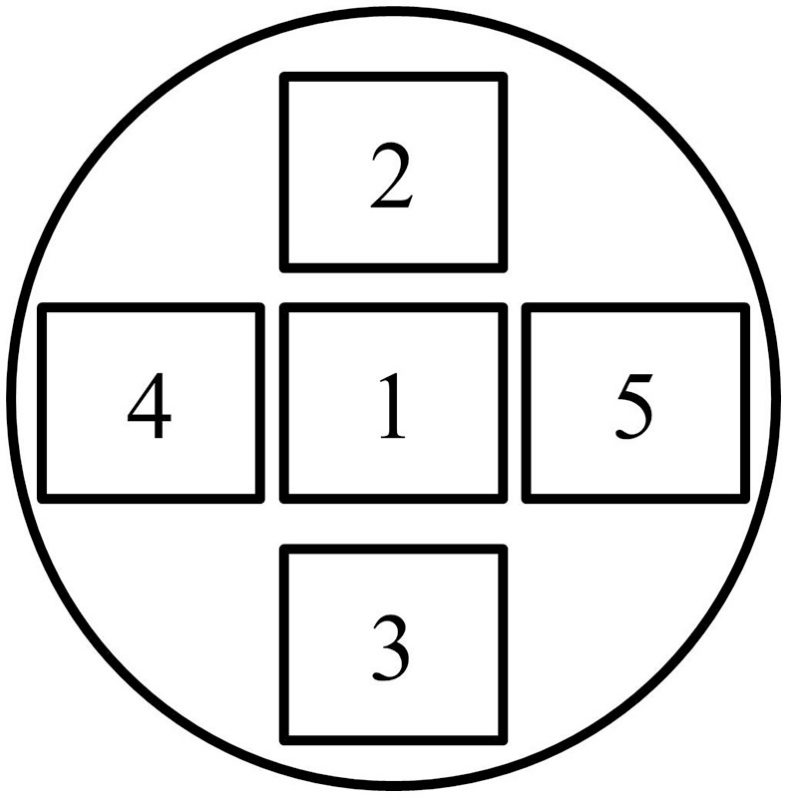
Overview of the five standardized positions for making images in the immunocytochemistry samples.

All immunocytochemistry samples were photographed with the Leica DM6B upright microscope (Leica Micro Systems Inc., Buffalo Grove, IL, USA) and Leica Application Suite X software (LASX, Leica Micro Systems Inc.). Images were made at a magnification of 70x. Five images per sample were taken to obtain a representative impression of each sample. This procedure was standardized to five positions (Figure 1). In order to make a 3D image, multiple Z-stacks were made for every sample, ranging from 30 to 75 steps (0.2 μm/step). The images were automatically optimized for brightness and contrast using the NIH ImageJ software. Subsequently, maximal intensity images were created from 15 to 20 steps in regions where microvilli and membrane stainings were most obvious. After critical reflection, a representative image was selected for each condition. For CLDN7 and actin filaments, an additional movie was made of all photographic steps using the NIH ImageJ software at 7 frames per second.

### Statistics

The average values are presented as mean ± SD. All statistical analyses were performed in Graphpad Prism (Version 6.07). Student *T*-tests were applied to assess changes in OCR and the different OXPHOS complex protein levels between Caco-2 cells cultured in DMEM-glucose and those cultured in DMEM-galactose. Student *T*-tests were also applied to assess changes in LDH release, cellular ATP levels, fluorescein flux, and tight junction gene expression between Caco-2 cells treated with CCCP, PA or ROT and their corresponding controls. Two-way ANOVA with Sidak's multipal comparisons was applied to compare changes in *SIM* gene expression between the differentiation status and energy substrate (glucose vs. galactose). In case data were not normally distributed, data were log transformed (*TJP1, TJP2*, and *CLDN2*). Gene expression of *TJP1* was also after log transformation not normally distributed and a Mann Whitney *U*-test was performed instead. One-way ANOVA with Sidak's multiple comparisons *post hoc* testing was applied to assess differences in sucrase-isomaltase gene expression between Caco-2 cells differentiated in DMEM-glucose and those differentiated in DMEM-galactose. Statistical significance was defined as a two-tailed *p* < 0.05.

## Results

### Increased oxidative metabolic phenotype of Caco-2 cells cultured in DMEM-galactose

To establish the relation between mitochondrial ATP production and intestinal permeability it is essential that ATP production in the culture system is dependent on mitochondria and not on glycolysis, which is seen in cells cultured in glucose-rich medium. Therefore, we first investigated whether a switch to high-galactose (25 mM) medium (DMEM-galactose) stimulates mitochondrial ATP production in Caco-2 cells as described previously for other cell types (Rossignol et al., 2004).

**Figure 2:**
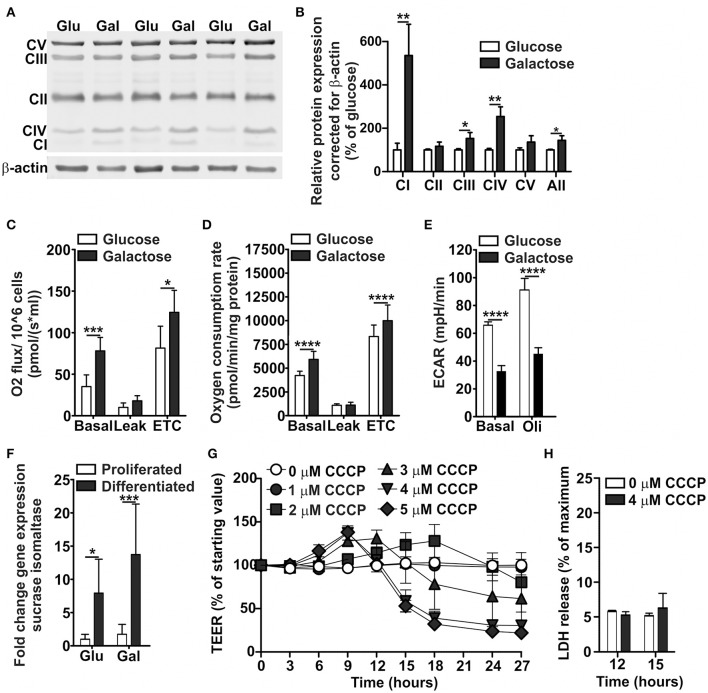
Oxidative metabolic characterization of Caco-2 cells proliferated and differentiated in glucose- and galactose-containing medium. Caco-2 cells were proliferated in DMEM-glucose or DMEM-galactose for 10 days, after which **(A,B)** levels of OXPHOS complexes, and **(C)** basal, leak, and maximal (electron transport chain, ETC) oxygen consumption rates were determined. Caco-2 monolayers were semi-differentiated in DMEM-glucose and DMEM-galactose for 14 days after which **(D)** basal, leak, and maximal oxygen consumption rates and **(E)** basal and oligomycin (Oli)-induced ECAR values were determined. 14-days differentiated Caco-2 monolayers grown in DMEM-glucose and DMEM-galactose were tested for **(F)** their sucrase-isomaltase gene expression; Gene expression was normalized with the mRNA gene expression of β-actin and β-2 microglobulin. The 14-days differentiated Caco-2 monolayers using DMEM-galactose were incubated with CCCP (0-1-2-3-4-5 μM) after which **(G)** the transepithelial electrical resistance (TEER) and **(H)** the cytotoxicity, as assessed by the release of lactate dehydrogenase (LDH), were followed over time. The results are expressed as a mean ± SD. The ^*^ symbol indicates statistical significance of *P* < 0.05, ^**^P < 0.01, ^***^P < 0.001, and ^****^P < 0.0001.

Visualization of the mitochondria suggested an increase in mitochondrial density and network branching when Caco-2 cells were grown on DMEM-galactose as compared to DMEM-glucose (Figure S1). In line with this finding, protein levels of subunits of the OXPHOS complexes I, III, and IV were significantly increased on DMEM-galactose compared to DMEM-glucose (Figures 2A,B). The largest increase was seen for complex I, followed by complex IV and III. Functionally, Caco-2 cells grown on DMEM-galactose showed significantly increased basal and maximal OCR. These effects were similar for both undifferentiated Caco-2 cells, as measured with the Oroboros high-resolution oxygraph (Figure 2C), and semi-differentiated Caco-2 cells, as measured with the Seahorse XFe96 (Figure 2D). These semi-differentiated cells grown on DMEM-galactose showed ECAR values that were decreased to about half the values as seen when they were grown on DMEM-glucose (Figure 2E). Caco-2 cells differentiated on DMEM-galactose showed higher absolute TEER values (approximately 300 vs. 200 Ω·cm^2^). There was no significant effect of substrate (glucose vs. galactose) on the gene expression of the brush border enzyme sucrase-isomaltase, but there was a significant effect of differentiation status (about 8-fold increase for glucose vs about 14-fold increase for galactose, Figure 2F).

To provide a first link between mitochondrial ATP production and Caco-2 monolayer integrity, indicated by TEER values, we uncoupled the mitochondria using CCCP, which is also used to determine maximal OCR. CCCP dose-dependently decreased TEER in 14-days differentiated Caco-2 cell monolayers when cultured in DMEM-galactose after 12 h of incubation (Figure 2G). At 12 and 15 h of CCCP incubation, LDH release was not increased (Figure 2H), indicating that the major decrease in TEER seen after 15 h could not be explained by cytotoxicity.

Altogether, these findings indicate that Caco-2 cells cultured in DMEM-galactose switched to an oxidative metabolic phenotype and became dependent on mitochondrial ATP production, and inhibition of mitochondrial ATP production resulted in decreased Caco-2 monolayer integrity or, in other words, increased Caco-2 permeability. Although, CCCP induces mitochondrial OXPHOS uncoupling, it may exert non-mitochondrial effects in the cell (Lichtshtein et al., 1979; Gottlieb et al., 2003). Therefore, we decided to specifically obstruct mitochondrial ATP production by inhibiting complex I, the OXPHOS complex that showed the largest increase in protein levels when Caco-2 culture medium was switched from DMEM-glucose to DMEM-galactose.

### Rotenone and piericidin A increase permeability and decrease cellular energy status of Caco-2 monolayers differentiated in DMEM-galactose

**Figure 3:**
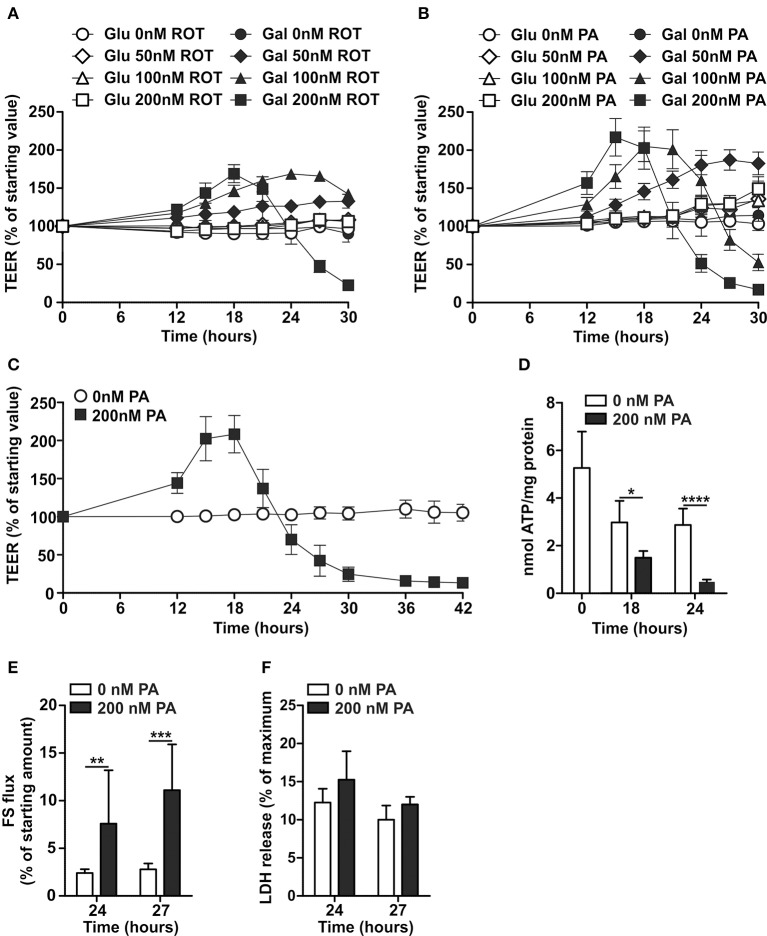
Effects of Complex I inhibition on differentiated Caco-2 cell monolayers cultured in glucose- and galactose-containing medium. Fourteen-days differentiated Caco-2 monolayers using DMEM-glucose (Glu) or DMEM-galactose (Gal) were incubated with vehicle (96% EtOH) or rotenone (ROT; 50–100–200 nM) or Piericidin A (PA; 50–100–200 nM) after which TEER was followed over time, **(A,B)** show representative examples of a single experiment and **(C)** the average of three independent experiments (only GAL ± 200 nM PA). Fourteen-days differentiated Caco-2 monolayers grown in DMEM-galactose were incubated with vehicle (96% EtOH) or 200 nM PA, after which **(D)** cellular ATP status, **(E)** fluorescein (FS) passage from the apical to the basolateral side of the monolayer, and **(F)** cytotoxicity by lactate dehydrogenase (LDH) release were determined. The results are expressed as a mean ± SD. The ^*^ symbol indicates statistical significance of *P* < 0.05, ^**^P < 0.01, and ^****^P < 0.0001.

ROT induced, in a concentration-dependent manner, first an increase in TEER followed by a decrease in TEER of Caco-2 monolayers when cultured in DMEM-galactose, but not DMEM-glucose (Figure 3A). However, ROT has been shown to have the potency to destabilize microtubules (Marshall and Himes, 1978), which are critical in the maintenance tight junctions and thus the integrity of the epithelial barrier (Glotfelty et al., 2014). These off-target effects have not been related to PA, which was therefore used in the remainder of this study. Similar to ROT, PA induced in a concentration-dependent manner first an increase and then a decrease in TEER of Caco-2 monolayers cultured in DMEM-galactose, but not in DMEM-glucose (Figure 3B). The lowest concentration of PA of 50 nM was insufficient to induce a decrease in TEER, while 100 nM of PA resulted in a delayed but similar pattern as 200 nM of PA, which showed the largest effects on Caco-2 monolayer permeability without observed cytotoxicity (Figure 3F). The addition of 200 nM of PA furthermore resulted in a significant reduction in the cellular energy status as reflected by decreased levels of ATP, already at the point where TEER was still increased, but more pronounced at the point where TEER was decreased (Figures 3C,D). In contrast, ATP levels in Caco-2 cells cultured in DMEM-glucose, were not affected by 200 nM PA after 24 hours (control mean ± SD: 4.6 ± 2.7 nmol ATP/mg protein; 200nm PA mean ± SD: 4.5 ± 0.4 nmol ATP/mg protein). This decrease in TEER of 200 nM PA-treated Caco-2 monolayer was accompanied by increased fluorescein flux over the monolayer from the apical to basolateral compartment (Figure 3E).

These results suggest that an increased permeability of the oxidative Caco-2 monolayer is a result from a decreased cellular energy status by inhibition of mitochondrial ATP production.

### Inhibition of mitochondrial ATP production affects the expression of tight junction genes in oxidative Caco-2 monolayers

**Figure 4:**
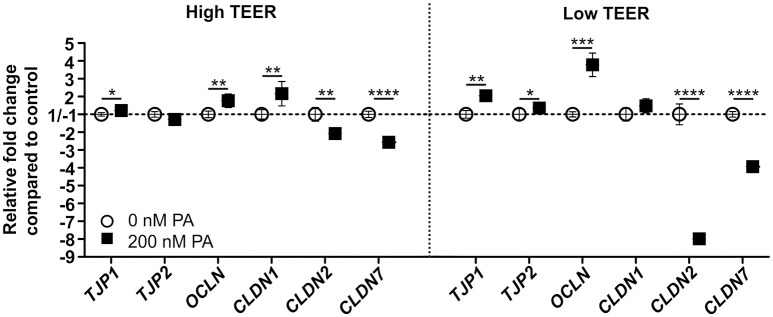
Gene expression of members of the tight junctions in differentiated Caco-2 cell monolayers upon PA treatment. Fourteen-days differentiated Caco-2 monolayers using DMEM-galactose were incubated with 0 nM (vehicle; 96% EtOH) or 200 nM Piericidin A (PA), after which mRNA gene expression of tight junction protein 1 (*TJP1*), tight junction protein 2 (*TJP2*), occludin (*OCLN*), claudin 1 (*CLDN1*), claudin 2 (*CLDN2*), and claudin 7 (*CLDN7*) were determined when the transepithelial resistance (TEER) of the monolayer was high (High TEER) and low (Low TEER). Gene expression was normalized with the mRNA gene expression of the β-2 microglobulin (*B2M*), transmembrane protein 14C (*TMEM14C*), and glutaminyl-tRNA synthetase (*QARS*). The ^*^ symbol indicates statistical significance of *P* < 0.05, ^**^P < 0.01, ^***^P < 0.001, and ^****^P < 0.0001.

mRNA gene expression of tight junction genes was analyzed at the point of the clear increase (150–200%) and the clear decrease (40–60%) in TEER of the DMEM-galactose-cultured Caco-2 monolayers treated with 0 (control) or 200 nM PA. When the TEER was high (~15 h of PA treatment; Figure 3C), gene expression levels of tight junction protein 1 (*TJP1*; *P* = 0.04), occludin (*OCLN*; *P* = 0.001) and claudin 1 (*CLDN1*; *P* = 0.003) was significantly increased upon PA treatment as compared to control. In contrast, gene expression levels of tight junction protein 2 (*TJP2*) was unchanged, while the expression of claudin 2 (*CLDN2*; *P* = 0.003) and claudin 7 (*CLDN7*; *P* = < 0.0001) were significantly decreased upon PA treatment compared to the control (Figure 4). These outcomes were at low TEER (~24 h of PA treatment; Figure 3C) more pronounced for *TJP1* (*P* = 0.002), *OCLN* (*P* = < 0.0001), *CLDN2* (*P* = < 0.0001), and *CLDN7* (*P* = < 0.0001). However, at this point the increased gene expression of *CLDN1* showed merely a trend (*P* = 0.06), while gene expression of *TJP2* became significantly increased (*P* = 0.02) upon PA treatment as compared to the control (Figure 4).

### Inhibition of mitochondrial ATP production results in membrane-to-cytoplasmic relocation of claudin 7 in oxidative Caco-2 monolayers

**Figure 5:**
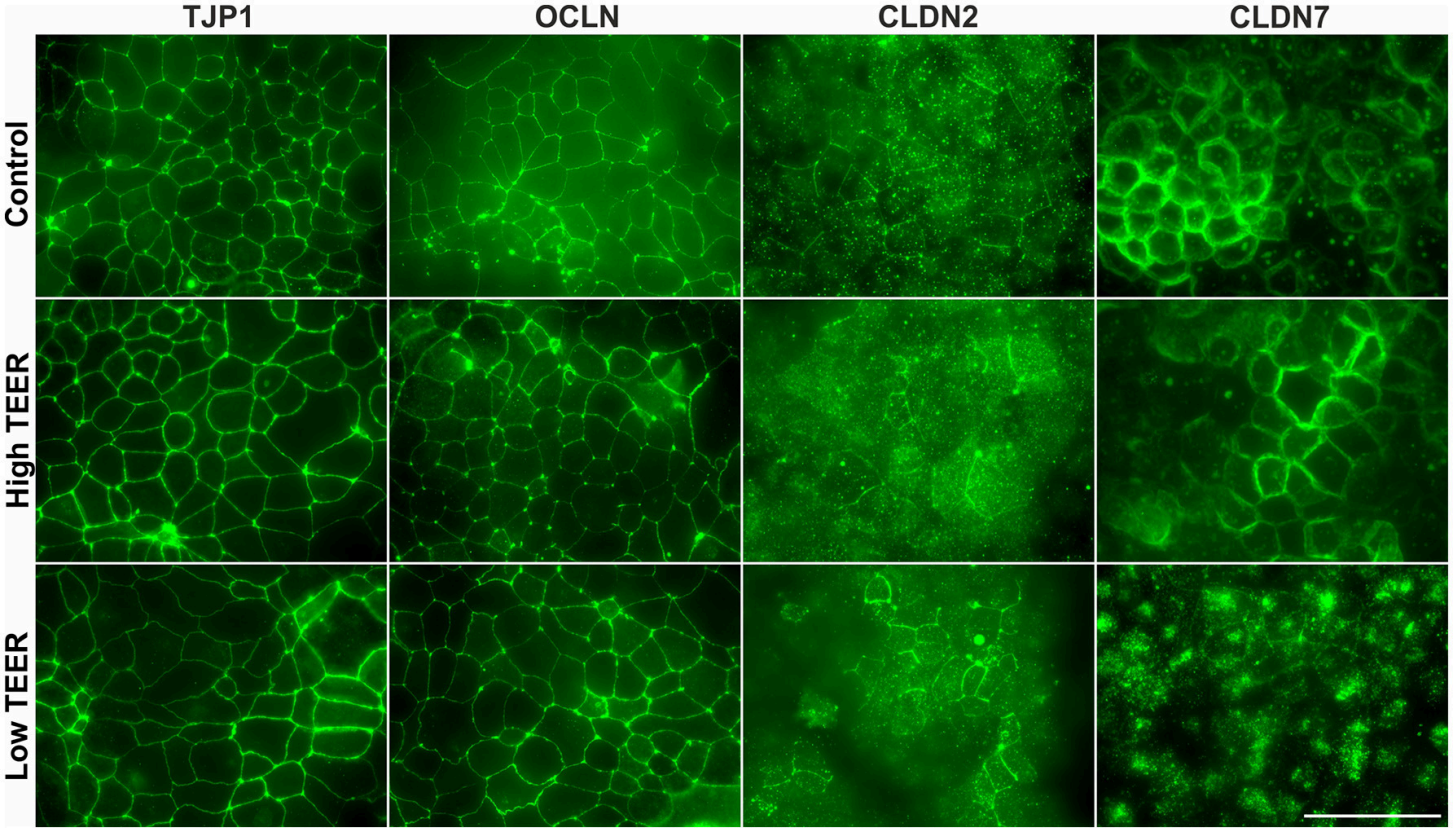
Distribution of tight junction proteins in differentiated Caco-2 cell monolayers upon PA treatment. Fourteen-days differentiated Caco-2 monolayers using DMEM-galactose were incubated with vehicle (96% EtOH, control) or 200 nM Piericidin A (PA), after which immunofluorescent staining was performed on tight junction protein 1 (TJP1), occludin (OCLN), claudin 2 (CLDN2), and claudin 7 (CLDN7). Changes in protein distribution were analyzed in maximal intensity images created from 15 to 20 steps in regions where membrane stainings were most obvious at the moments that the transepithelial resistance (TEER) of the monolayer was high (High TEER) and low (Low TEER). Scale bar represents 50 μm.

The distribution of different proteins involved in tight junction maintenance are shown in Figure 5 at the point of a clear increase (150–200%) and the clear decrease (40–60%) in TEER of the DMEM-galactose-cultured Caco-2 monolayers treated with 0 (control) or 200 nM PA. In the control samples both tight junction protein 1 (TJP1) and occludin (OCLN) were found in the cell membrane and specifically just below the microvilli (based on actin results). This specific localisation remained unchanged when the TEER was high or low. Claudin 2 (CLDN2) was also found to be located just below the microvilli, but only partly in the surrounding cell membrane. When TEER was high and especially low, more CLDN2 appears to be located in the cell membrane just below the microvilli. Claudin 7 (CLDN7) was located along the cell membrane spread from the apical to basolateral side in the control Caco-2 monolayers and not just below the microvilli as seen for TJP1, OCLN, and CLDN2. At high TEER a similar CLDN7 distribution was found, but at low TEER CLDN7 was almost completely relocated from the cell membrane into the cytoplasm (see also supplementary movie 2).

### Inhibition of mitochondrial ATP production results in reduced microvilli density and actin protein distribution in oxidative Caco-2 monolayers

**Figure 6:**
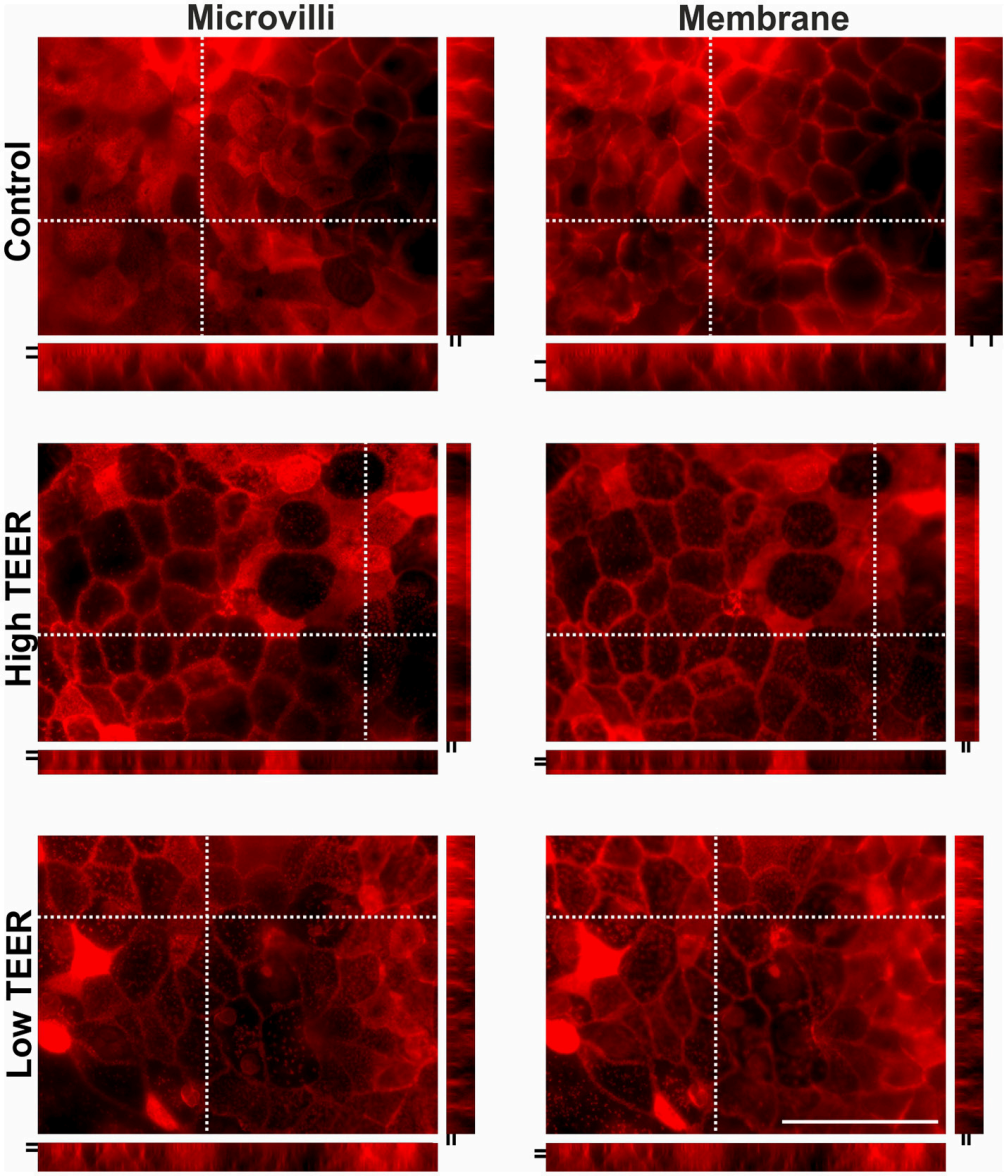
Actin protein staining in differentiated Caco-2 cell monolayers upon PA treatment. Fourteen-days differentiated Caco-2 monolayers grown in DMEM-galactose were incubated with vehicle (96% EtOH, control) or 200 nM Piericidin A (PA), after which actin immunofluorescent staining was performed. Changes in protein distribution reflecting microvilli length and membrane structure were analyzed in maximal intensity images created from 15 to 20 steps in regions where membrane stainings were most obvious at the moments that the transepithelial resistance (TEER) of the monolayer was high (High TEER) and low (Low TEER). Dashed crossed line in the XY images marks the position of the XZ (below the XY image) and YZ (right side of XY image) side views. The space between the two short black lines in the XZ and YZ side views represent thickness of microvilli and relative membrane thickness, respectively. Scale bar represents 50 μm.

Actin protein staining as shown in Figure 6 (see also supplementary movie 1) was performed at the point of a clear increase (150–200%) and the clear decrease (40–60%) in TEER of the DMEM-galactose-cultured Caco-2 monolayers treated with 0 (control) or 200 nM PA. When TEER was high and low the density of microvilli, as observed in the XY dimension, was decreased as compared to control, but no large changes in length of the microvilli was seen, as observed (and indicated by the black lines) in the XZ and YZ dimension. Furthermore, the ring structure of actin protein surrounding the cell membrane was much thinner and less abundant, as observed (and indicated by the black lines) in the XZ and YZ dimension, as compared to the control.

## Discussion

In this study, we newly developed a Caco-2 cellular model that was dependent on mitochondrial ATP production for its energy requirements. To achieve this, a substrate switch from DMEM-glucose to DMEM-galactose was applied, based on previous experiments with other cell lines (Rossignol et al., 2004; Marroquin et al., 2007; Aguer et al., 2011; Kase et al., 2013). This oxidative Caco-2 monolayer model provides a condition more similar to the *in vivo* situation, which is characterized by high oxygen dependency as observed in studies of ischemia (Grotz et al., 1999; Hinnebusch et al., 2002; Grootjans et al., 2010). Inducing mitochondrial dysfunction by either uncoupling or inhibition of OXPHOS function resulted in decreased cellular ATP levels followed by decreased TEER and increased paracellular flux of fluorescein. These findings indicate that maintenance of intestinal barrier function is highly dependent on mitochondrial ATP production.

A relation between cellular ATP levels and cell monolayer permeability was also observed in other studies (Bacallao et al., 1994; Unno et al., 1996; Tsukamoto and Nigam, 1997; Gopalakrishnan et al., 2007; Wagner et al., 2008). However, in these studies epithelial cell monolayers were cultured in high glucose conditions making them highly glycolytic and independent of mitochondrial ATP production, while our study uses oxidative conditions representing the *in vivo* situation. In these glycolytic epithelial cell monolayers 2-deoxyglucose, inhibiting glycolysis, or a combination of 2-deoxyglucose and antimycin A, inhibiting both glycolysis and OXPHOS, resulted in a fast and severe decrease in cellular ATP levels as well as an increase in monolayer permeability (Bacallao et al., 1994; Unno et al., 1996). Similar results were also obtained under glycolytic conditions using indomethacin, with or without ROT, both of which have been shown to inhibit OXPHOS Complex I, thus targeting mitochondria (Carrasco-Pozo et al., 2011). However, a fast, within minute, increase in permeability was induced, which is in sharp contrast to our study which showed no decrease in cellular ATP in glycolytic Caco-2 cells and a gradual dose-dependent response in oxidative Caco-2 monolayers. The rapid permeability decrease can be due to the very high concentrations of OXPHOS inhibitors used in the other studies, far exceeding (>200 times) our minimal concentrations of OXPHOS inhibitors that maximally reduced cellular ATP status (Bacallao et al., 1994; Carrasco-Pozo et al., 2012). Such high doses have been shown to induce severe off-target effects such as destabilization of microtubules (Marshall and Himes, 1978), high levels of oxidative stress (Carrasco-Pozo et al., 2012), and Rho-GTPase-induced changes in tight junction or cytoskeleton structure (Sanchez et al., 2008; Grefte et al., 2015). Still, ROT can show off-target effects at very low concentrations and thus we decided to also include PA, which does not display these off-target effects (Grefte et al., 2015). Additionally, the absence of any effect of ROT and PA in the Caco-2 monolayers under glycolytic conditions indicate that both ROT and PA at these doses had no such off-target effects.

To obtain a first insight in the mechanisms underlying the decrease in TEER, we analyzed the expression of several tight junction genes in the oxidative Caco-2 monolayers incubated with PA. Since functionality will be mainly determined by changes in levels and localisation of the different proteins, also the distribution of the corresponding proteins in relation to monolayer permeability was analyzed (Price et al., 2014). There are many proteins involved in the formation of the tight junctions, serving various functions. The TJPs together with OCLN mainly support and stabilize the structure of the tight junctions (Van Itallie et al., 2009).

The observed increases in gene expression of *TJP1, TJP2*, and *OCLN* suggest a compensatory mechanism of the Caco-2 cells, in an attempt to stabilize and protect the structure of the tight junctions (Zeissig et al., 2007). In the current study, however, no effect on the levels or distribution of TJP1 and OCLN was observed for Caco-2 monolayers treated with PA. This differs from other studies that show an effect on the localisation of these proteins in antimycin A or rotenone-induced ATP depleted Caco-2, MDCK, and glomerular epithelial cell monolayers (Gopalakrishnan et al., 2007; Wagner et al., 2008; Carrasco-Pozo et al., 2013). Since the proteins that form the tight junctions are closely associated with the cytoskeleton (Tsukamoto and Nigam, 1997), the found effects in the ladder studies, which employ high amounts of compounds, could possibly be caused by indirect effects rather than decreased ATP levels. For our studies we show that the PA treatment has an effect on the abundance of the actin ring structure surrounding the cell membrane, but this does not seem to be directly related to the observed changes in TEER.

In contrast to the TJPs and OCLN, the CLDNs have shown to play an essential role in the actual paracellular permeability, either by tightening junctions or by forming pores that open the paracellular pathway to selective molecules (Lee, 2015). CLDN1 and CLDN7 have mainly been described to tighten the junctions (Alexandre et al., 2005; Hou et al., 2006; Tanaka et al., 2015), while CLDN2 can form paracellular channels for cations (Na^+^) and water and is therefore associated with a “leaky” barrier (Amasheh et al., 2002; Hou et al., 2006; Rosenthal et al., 2010, 2017; Luettig et al., 2015). The upregulation of *CLDN1* and downregulation of *CLDN2* in this study suggest a first defense mechanism to prevent an increase in intestinal permeability. The downregulation of the gene expression of “tightening” *CLDN7*, on the other hand, is not consistent with such a protective response. For CLDN2 and CLDN7 there were, however, also changes observed for protein localisation. CLDN2 seemed to be slightly increased on the apical membrane upon PA treatment which coincided with a decrease in TEER possibly resulting from increased pore formation. This is in line with earlier findings in MCDK-II cells (Hou et al., 2006), but also relates to intestinal diseases such as Crohn's disease and celiac disease showing increased CLDN2 protein levels (Zeissig et al., 2007; Weber et al., 2008; Luettig et al., 2015).

Crucially, a clear membrane-to-cytoplasm redistribution of CLDN7 was found at low TEER whereas this was not seen in control or high TEER, suggesting that loss of membranous CLDN7 is responsible for the decreased Caco-2 monolayer integrity. This is in line with the role of CLDN7 in tightening the tight junctions and that a deficiency of CLDN7 in mice is associated with inflammation and decreased intestinal integrity (Alexandre et al., 2005; Hou et al., 2006; Ding et al., 2012; Tanaka et al., 2015). CLDN7 is present throughout the intestine (Fujita et al., 2006; Holmes et al., 2006) and it is found to form a paracellular barrier to Na+ and a channel for Cl-, depending on cell type (Hou et al., 2006). Next to that, CLDN7 is proposed to have a function not related to tight junctionss because of its localisation on the basolateral and not apical side of the lateral membrane at the level of the tight junctions (Ding et al., 2012), which is supported by our findings showing an apical-to-basolateral distribution of CLDN 7 in the membrane of Caco-2 cells. This suggests that CLDN7 plays an important role in maintaining intestinal integrity. Our findings are in accordance with Hou et al. (2006), who also found that a loss of CLDN7 resulted in a decreased TEER.

Surprisingly, our studies showed first a dose-dependent increase in the TEER, followed by a similar dose-dependent decrease in TEER, independent of whether the Caco-2 monolayers were treated with CCCP, ROT, or PA. We speculate that since TEER is based on ion permeability of the paracellular pathway (Ranaldi et al., 2003), it may indicate any physiological response of the cells that limits ion fluxes. The increase in TEER could reflect an initial defense mechanism of the intestinal epithelial cells to energy stress including redistribution of tight junction proteins (Van Itallie et al., 2008), although this is not supported by our findings. Downregulation of *CLDN2* suggests indeed a decreased monolayer permeability, but this is not supported by the slight increase in its protein levels at the cell membrane, which suggests the opposite. The localization of TJP1, OCLN, and CLDN7 remained unchanged. Other mechanisms may also play a role, including possible shutdown of ATP-dependent ion pumps (Bacallao et al., 1994; Ranaldi et al., 2003). The latter may be part of a cellular energy conservation strategy. For example, Na^+^, K^+^-ATPase constitutes the major component of basal metabolic rate (Rolfe and Brown, 1997), is fuelled by mitochondrial ATP (Fernández-Moncada and Barros, 2014) and has been indicated to play a role in tight junction formation in epithelial cells (Rajasekaran et al., 2001). Moreover, AMPK, the major cellular energy sensor, has been implicated in barrier function (Peng et al., 2009; Wang et al., 2016) and the increase in TEER may be due to alterations in AMPK activity.

It can be concluded from our studies that mitochondria play an essential role in maintaining an intestinal barrier with high integrity. This is supported by the dependency on mitochondrial ATP production of Caco-2 monolayers grown on DMEM-galactose, thereby mimicking the *in vivo* situation by showing a more oxidative metabolic phenotype that is responsive to mitochondrial dysfunction. Changes in Caco-2 monolayer permeability may be facilitated by increased CLDN2 at the tight junctions and, more importantly, a membrane-to-cytoplasm redistribution of CLDN7. The intestine is very sensitive to a decrease in supply of oxygen (ischemia) or an increase in demand (Zeitouni et al., 2016), underlining that our DMEM-galactose cultured Caco-2 model provides a physiologically more relevant (oxidative) phenotype to study the mechanism underlying intestinal permeability as observed during *in vivo* situations. Next to normal physiological conditions, such as endurance exercise, intestinal permeability is also observed in many disease-related situations (Bjarnason et al., 1995). It is attractive to think that studying intestinal permeability in detail using our oxidative Caco-2 model could potentially offer therapeutic targets to improve intestinal disease states.

## Author contributions

LJ, SG, DD, KvN, HW, and JK concepted and designed the work, LJ, SG, VdB, LZ, IvS, MB, and JK contributed in acquisition, analysis, or interpretation of the data, LJ, SG, and JK drafted the manuscript, while the other authors critically revised the manuscript. All authors approved the final version of the manuscript for publication and agree to be accountable for all aspects of the work in ensuring that questions related to the accuracy or integrity of any part of the work are appropriately investigated and resolved.

### Conflict of interest statement

The authors declare that the research was conducted in the absence of any commercial or financial relationships that could be construed as a potential conflict of interest.

## Supplementary material

Figure S1 Oxidative metabolic characterization of Caco-2 cells proliferated and differentiated in glucose- and galactose-containing medium. Caco-2 cells were proliferated in DMEM-glucose or DMEM-galactose for 10 days, after which mitochondrial structures were visualized with the EVOS FL Color Imaging System. Pictures were taken using a 60x objective, scale bar represents 50 μm for both pictures).

Supplementary Movie 1 Actin protein staining in differentiated Caco-2 cell monolayers upon PA treatment. Fourteen-days differentiated Caco-2 monolayers grown in DMEM-galactose were incubated with vehicle (96% EtOH, control) or 200 nM Piericidin A (PA), after which actin immunofluorescent staining was performed. Changes in protein distribution reflecting microvilli length and membrane structure were analyzed in maximal intensity images created from 15 to 20 steps in regions where membrane stainings were most obvious at the moments that the transepithelial resistance (TEER) of the monolayer was high (High TEER) and low (Low TEER).

Supplementary Movie 2 Distribution of claudin 7 in differentiated Caco-2 cell monolayers upon PA treatment. Fourteen-days differentiated Caco-2 monolayers using DMEM-galactose were incubated with vehicle (96% EtOH, control) or 200 nM Piericidin A (PA), after which immunofluorescent staining was performed on claudin 7. Changes in protein distribution were analyzed in maximal intensity images created from 15 to 20 steps in regions where membrane stainings were most obvious at the moments that the transepithelial resistance (TEER) of the monolayer was high (High TEER) and low (Low TEER).
