# Supplementary figures and images for: Mitochondrial ATP Depletion Disrupts Caco-2 Monolayer Integrity and Internalizes Claudin 7
Source: Front Physiol. 2017 Oct 11;8:794. doi: 10.3389/fphys.2017.00794 (PMC5641570; doi:10.3389/fphys.2017.00794)

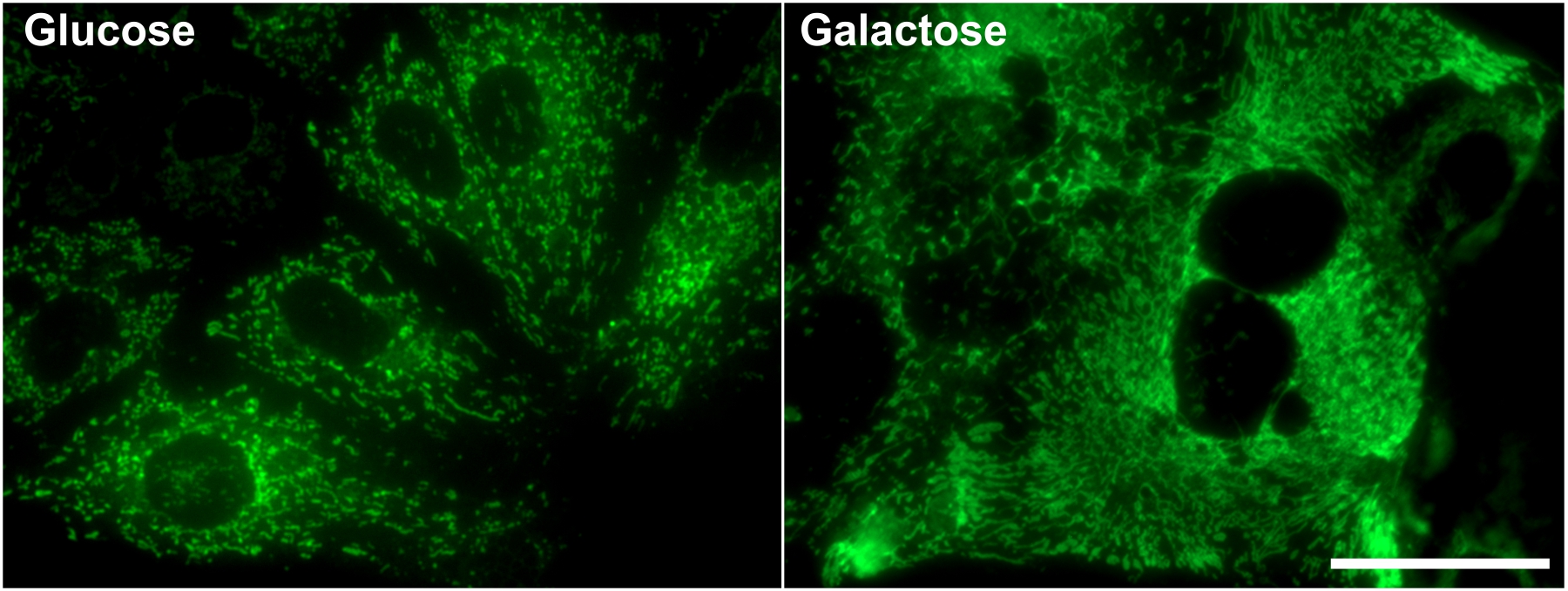

Supplement: Figure S1 — Oxidative metabolic characterization of Caco-2 cells proliferated and differentiated in glucose- and galactose-containing medium. Caco-2 cells were proliferated in DMEM-glucose or DMEM-galactose for 10 days, after which mitochondrial structures were visualized with the EVOS FL Color Imaging System. Pictures were taken using a 60x objective, scale bar represents 50 μm for both pictures). [file Image1.JPEG]
